# Metagenomics reveals contrasted responses of microbial communities to wheat straw amendment in cropland and grassland soils

**DOI:** 10.1038/s41598-025-98903-2

**Published:** 2025-04-27

**Authors:** Domitille Jarrige, Vincent Tardy, Valentin Loux, Olivier Rué, Abad Chabbi, Sébastien Terrat, Pierre-Alain Maron

**Affiliations:** 1 Agroécologie, INRAE, Université de Bourgogne, Institut Agro, Université de Bourgogne Franche-Comté, Dijon, F-21000 France; 2 Université Paris Saclay, INRAE, Jouy-en-Josas, MaIAGE France; 3 INRAE, BioinfOmics, MIGALE bioinformatics facility, Université Paris-Saclay, Jouy-en- Josas, France; 4 INRAE, Poitou-Charentes, URP3F, Lusignan, 86600 France; 5 UMR-ECOSYS Joint research unit INRAE, Université Paris-Saclay, AgroParisTech, Paris, France

**Keywords:** Agroecology, Soil microbiology, Metagenomics, Biodiversity

## Abstract

Soil microbial communities respond quickly to natural and/or anthropic-induced changes in environmental conditions. Metagenomics allows studying taxa that are often overlooked in microbiota studies, such as protists or viruses. Here, we employed metagenomics to characterise microbial successions after wheat straw input in a 4-month in-situ field study. We compared microbial successions patterns with those obtained by high throughput amplicon sequencing on the same soil samples to validate metagenomics as a tool for the fine analysis of microbial population dynamics in situ. Taxonomic patterns were concordant between the two methodologies but metagenomics allowed studying all the microbial groups simultaneously. Notably, our results evidenced that each domain displayed a specific dynamic pattern after wheat straw amendment. For instance, viral sequences multiplied in the early phase of straw decomposition, in parallel to copiotrophic bacteria, suggesting a “kill-the-winner” pattern that, to our knowledge, had not been observed before in soil. Altogether, our results highlighted that both inter and intra-domain trophic interactions were impacted by wheat amendment and these patterns depended on the land use history. Our study highlights that top-down regulation by microbial predators or viruses might play a key role in soil microbiota dynamics and structure.

**Supplementary Information:**

The online version contains supplementary material available at 10.1038/s41598-025-98903-2.

## Introduction

Soil ecosystems harbour complex and highly diverse microbial communities, comprising microorganisms from all domains of life: *Archaea*, *Bacteria*, *Eukaryota* (comprising *Fungi* and protists, i.e., a paraphyletic group including non-fungal, non-metazoan and non-land plant eukaryotes^1^) and *Viruses*. This microbiome responds strongly to land-use (e.g. forest, grassland, cropland)^2^ and agricultural practices such as tillage, fertilization, crop rotation and protection strategies^3,4^. These anthropogenic parameters significantly impact soil microbial diversity, with consequences in terms of soil sustainability and productivity, since soil functioning results from the activity of the huge diversity of these interacting microbial communities^5–8^. Such observations have stimulated much research to decipher the dynamic patterns of soil microbiota. Our ability to better manage microbial diversity and associated functions depends on our capacity to understand these dynamics.

The addition of crop residues to soils is a widespread agricultural practice aimed at enhancing soil organic matter (SOM) content, and improving soil structure^9,10^. As such, much effort was dedicated to determine the extent to which it changes the diversity and abundance of soil microbial communities. Studies have focussed on different questions such as the impact of residue quality^11,12^, quantity and localisation in the soil^13^. All these studies considerably expanded our knowledge of the ecology and dynamics of the heterotrophic microbial communities involved in C-transformations in soil. However, most of these studies targeted specific groups, mainly *Bacteria* and/or *Fungi*. Thus, they lacked exhaustiveness and did not provide comprehensive understanding of the dynamics of the whole soil microbiome. Such holistic data are however needed to gain insights into the interactions between taxonomic groups, such as competition, predation, parasitism and symbiosis, especially since less studied microbial predators belonging to protists and *Viruses* might be crucial in regulating the soil microbiome and associated activity^14,15^.

Technical limitations impeding access to the diversity of the whole soil microbiota may in part explain this knowledge gap. Indeed, the main methodology used in the literature to study soil microbial diversity remains targeted metagenomics (i.e., high throughput amplicon sequencing, also called metabarcoding)^16^. It provides resource and cost-effective data on the ecology of indigenous microbial communities, but it is limited to the genes that are targeted, and the taxonomic groups that include them (the most commonly surveyed groups are *Bacteria*, *Archaea* and *Fungi*^16^). This makes it unsuitable for obtaining the whole picture of the microorganisms representing every domain of life interacting together in soils. In contrast, the metagenomics approach (i.e., whole metagenome sequencing), which does not rely on amplifying specific markers, allows achieving a global assessment of the complete soil microbiome (i.e., not restricted to a given domain). It has already been applied successfully for studying the ecology of the soil microbial community in several studies^17–19^. However, applications to decipher the dynamic patterns of the microbial communities in situ remain scarce, particularly in the soil environment and comparisons between targeted and global metagenomic approaches are still required to fully validate the performances and/or limitations of metagenomics for such assessments. To date, a few attempts have been made to compare targeted and global metagenomic approaches. They involved diverse experimental designs and materials (polar biocrusts, environmental DNA, sedimentary ancient DNA, pollen)^20–24^. Some studies showed large differences in the taxonomic profiles obtained with the two methods^20,22,24^, whereas others showed closer results^21,23^. Given the disagreement between studies so far, making conclusions about the congruence of both approaches remains uncertain and additional studies are needed.

In this paper, we present the results of a comprehensive in-situ field study conducted to evaluate the influence of contrasted land use history (20 years cropland vs. 17 years grassland) on soil microbial successions occurring after wheat straw input. For each land use history, two treatments were applied: without (control) and with wheat straw amendment. Four sampling dates were analysed for a total of 48 soil samples (3 replicates×2 treatments×2 plots×4 dates). We employed metagenomics to characterize the whole soil microbial diversity (*Archaea*, *Bacteria*, *Eukaryota* and *Viruses*), and compared the results obtained for *Bacteria*, *Archaea* and *Fungi* with those previously obtained using high throughput amplicon sequencing on the same soil samples^25^. We expected the results obtained by amplicon sequencing and global metagenomic to display similar conclusions in terms of bacterial and fungal dynamics following wheat straw input. It was a prerequisite to validate the suitability of metagenomics for characterising the dynamics of the whole soil microbiome diversity in situ. We hypothesized that due to differences in ecological strategies between the different organisms, response dynamics following wheat straw amendment would vary between *archaea*, *bacteria*, *fungi*, protists and viruses. In addition, we expected dynamic patterns to depend on the contrasted land use histories of the soil. Altogether, these results would provide information about the drivers of each group in terms of trophic attributes and/or biotic interactions.

## Results and discussion

### Amplicon sequencing and metagenomics depict similar pictures of the structure of bacterial and fungal communities

**Fig. 1:**
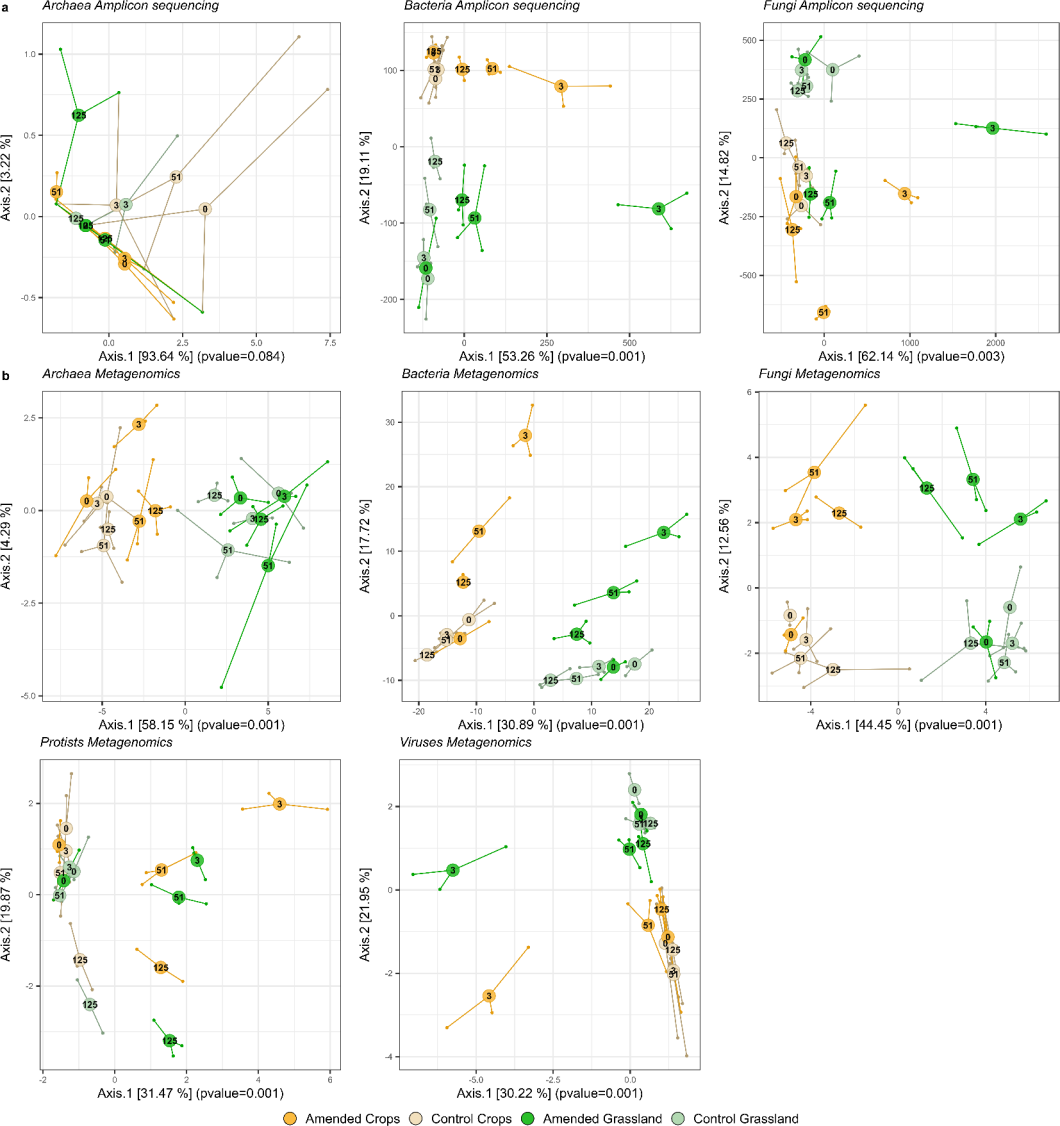
PCoA of communities separated by taxonomic group. Both metabarcoding (**a**) and metagenomics (**b**) separate communities according to the land-use history, and bacterial and fungal communities display similar patterns with both approaches. (**a**) PCoA of metabarcoding CLR transformed data. (**b**) PCoA of metagenomics CLR transformed taxonomic data. Green samples denote grassland, yellow crops, pale colours controls, deep colours amended samples. The percentage of explained variability for each axis is indicated in square brackets. The p-values of PERMANOVAs computed between cropland and grassland samples are indicated in parentheses on Axis.1.

The structures of bacterial and fungal communities obtained with amplicon sequencing^25^ and metagenomics displayed similar patterns in both control and amended treatments regardless of land-use history (Fig. 1). In addition, these two approaches provided similar results in terms of reproducibility, with the three field replicates showing close similarity (Fig. 1; Supplementary Fig. 1). Control grassland and cropland communities were discriminated on the factorial map, evidencing the impact of land-use history on the diversity of soil microbial communities^25,26^. In addition, while bacterial and fungal communities did not show any significant differences in their dynamics between the four sampling dates in the control, highlighting their stability along the seasons, the input of wheat residues strongly impacted these two communities, regardless of land-use history (Permutational Multivariate Analysis of Variance [PERMANOVA], *p*value < 0.001) (Supplementary Fig. 1). Thus, a strong population shift was observed after three days for both fungi and bacteria (Fig. 1). In the latter phase (days 51 and 125), bacterial communities but not fungal communities were resilient (Fig. 1).

Compared to *Bacteria* and *Fungi*, patterns of archaeal communities differed between the two sequencing approaches (Fig. 1). This discrepancy could be due to the lack of archaeal sequence recovered in the amplicon sequencing dataset. For this specific group, metagenomics proved to be far more resolutive than the metabarcoding dataset (probably due to its limited 4000 bacterial and archaeal sequences) and was able to detect a significant difference between cropland and grassland archaeal communities with PERMANOVA and Redundancy Analysis (RDA) (Supplementary Fig. 1 & Supplementary Table 1).

When looking at the relative abundance of the most abundant *phyla*, there was no major difference for bacterial and fungal *phyla* between amplicon sequencing and metagenomics (Supplementary Fig. 2), except for *Cyanobacteria* and *Basidiomycota* that were underrepresented in the amplicon sequencing datasets. In contrast, Becker and Pushkareva^20^ found that *Cyanobacteria* were overrepresented with their amplicon sequencing primers. The poor detection of *Cyanobacteria* in the amplicon sequencing dataset was also in apparent contradiction with previous in silico analyses^27^ of the PCR primers used in^25^, which predicted high hit frequency for *Cyanobacteria*. This could be explained by the change in taxonomic classification since the in silico analysis or by PCR biases in the in vivo application. This illustrates the potential drawback of amplicon sequencing whose representativeness for specific groups is highly dependent on the primers used and the PCR conditions. Moreover, thanks to its considerable depth of coverage, metagenomics identified many low abundance bacterial *phyla* that could not be detected with amplicons, among which were numerous candidate *phyla* (Supplementary Fig. 3).

At a finer taxonomic level, we compared the variations of raw count abundances of *genera* previously highlighted in^25^ during the experiment, with those obtained with the global metagenomic approach (Supplementary Fig. 4). For bacterial *genera*, the abundance patterns were consistent between the two methods (Supplementary Fig. 4a. and c.), with *genera* such as *Pseudomonas* and *Massilia* highly stimulated by straw amendment, regardless of land-use history. However, metagenomics appeared more sensitive than amplicon sequencing for some *genera* such as *Bulkholderia* and *Lysobacter*. Indeed, it showed a significant influence of straw amendment in both land-use histories for these *taxa*, whereas amplicon sequencing only detected a significant influence in one soil management history (Supplementary Fig. 4a. and c.). For fungal *genera*, both approaches also exhibited close abundance profiles (Supplementary Fig. 4b. and d.). Again, metagenomics appeared more sensitive than amplicon sequencing for certain *genera* such as *Rhizopus* and *Mucor*, for which hardly any sequences were detected in the control samples with amplicon sequencing. This could be explained easily by the much higher sequencing depth in metagenomics than in amplicon sequencing, as previously suggested^20^.

Overall, these results provide evidence that amplicon sequencing and metagenomics identified similar taxonomic patterns at the community, *phyla* and *genus* levels, highlighting that metagenomics is suitable for assessing the dynamics of the soil microbial communities in situ. Moreover, metagenomics was more sensitive than the amplicon dataset, showing the huge potential of this approach for the fine analysis of population dynamics. The similarity of the results observed between the two approaches for bacteria and fungi makes us confident about the robustness of the patterns observed for the other microbial groups (i.e., *Viruses* and protists) through metagenomics.

### Metagenomics revealed the temporal response to wheat input of the whole soil microbiota

**Fig. 2:**
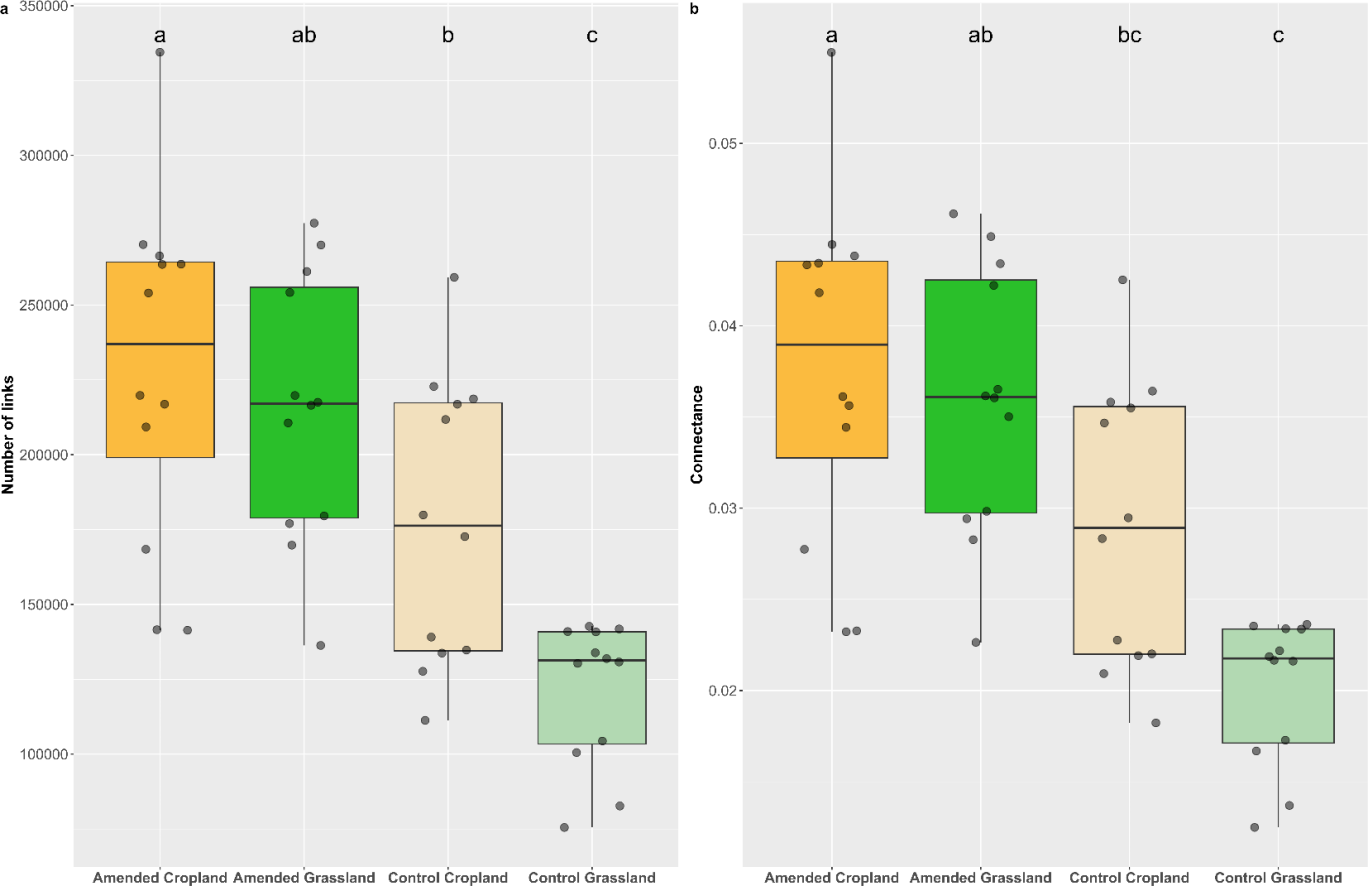
Cooccurrence networks statistics according to treatment and land-use history. Complexity of the whole microbial communities was impacted by land-use history and amendment, with more complexity in the cropland soil than in the grassland soil and a significative increase in complexity for both land-uses after wheat straw amendment. (**a**) Number of links. (**b**) Connectance (proportion of links formed out of all possible links). Kruskal Wallis tests with Bonferroni corrections (alpha 0.05) were performed on the metrics to assess their difference between different treatments and land-uses.

Metagenomics gave access to the whole soil microbiota, including *Viruses*, which is difficult to achieve with amplicon sequencing^28^. As with *Archaea*, *Bacteria* and *Fungi*, viruses and protists presented distinct structures between grasslands and croplands (Fig. 1b., PERMAVOVA, p-value < 0.001), showing a strong influence of land-use history. In addition to determining the structure of all microbial groups, land-use history also impacted the complexity of the whole soil microbial community, as evidenced by the results of the multigroup cooccurrence network analysis that revealed an increase of the total number of links and connectivity in cropland control compared to grassland control (Fig. 2). This result contradicts previous reports from national^29^ and regional scale studies^30^ that showed higher complexity of microbial networks in semi-natural systems such as forest and grassland compared to cropland. However, it must be kept in mind that our study deals with the effect of land-use history rather than with the land-use itself, since plant cover was removed and the soil was left bare by manual weeding for 5 months to stabilise before applying wheat residue inputs (see material and methods section). It is likely that such elimination of plant cover may have deeply affected the complexity of the soil microbial network, in agreement with^31^ who observed a high overall network complexity in the rhizosphere of oat that increased as plants grew. In other respects, this highlights the importance of plant cover for stimulating the complexity of soil microbial interactions and suggests that this effect is transient and disappears quickly following plant removal. In absence of plant cover, the lasting differences in soil properties (i.e. lower pH and phosphorous content; higher values of SOM, soil organic carbon content, total nitrogen, Soil C/N and cation exchange capacity in grassland soil) probably accounted for a large part of the microbial community’s differentiation between grassland and cropland soils (25).

As observed in the previous section for *Bacteria* and *Fungi*, wheat residue input strongly impacted the structure of *Viruses* and protists (Fig. 1 & Supplementary Table 1). Interestingly, it also significantly increased the complexity of the whole soil microbial community, as evidenced by the higher number of links and higher connectance of the multigroup microbial co-occurrence networks observed in both land-use histories (Fig. 2a, b.). This increase in the complexity of the soil microbial community by crop residue input had been reported previously for *Bacteria* and *Fungi*^32^, but to our knowledge, this is the first time it has been evidenced at the whole soil microbial community level. It points to the overall high network complexity that increases as C-inputs feed the soil microbiota, reflecting extensive interactions such as mutualism, competition, predation and parasitism that occur among microbial groups during the decomposition process.

The rapid and highly significant changes in the structure of both bacterial and fungal communities on day 3 after straw input (Fig. 1) reflected that not only bacteria but also fungi can act as pioneer decomposers of wheat straw^25,33^. The resilience of bacterial populations in the late phase of straw decomposition, where easily decomposable C-substrate was depleted, could be attributed to the decrease of copiotrophic bacteria populations (Fig. 1). In contrast, the different trajectories of the fungal communities implied that other fungal populations rose at days 51 and 135. Indeed, in this latter stage of straw decomposition, mainly recalcitrant organic carbon remained, thus fungal oligotrophs were likely preponderant, as was suggested before^25,33^ due to improved abilities to decompose more complex C-substrates compared to bacteria.

In amended plots, viral communities experienced transient but strong modifications on day 3 (Supplementary Fig. 1). Since *Viruses* are obligate parasites, such modifications can result only from the increase of their hosts abundance. Indeed, Virus would multiply through lysogenic or lytic infections. In oceans, viral lysis is estimated to turnover ~ 20% of microbial biomass every day and is a major actor of biogeochemical cycles by liberating nutrients and carbon^34^. In soil, *Viruses* have been studied less so far and very little is known about their roles in regulation of other microbial groups and carbon decomposition^35^. In our study, their short-lived response following wheat straw input might indicate a strong link with copiotrophic populations. They might be in part responsible for the rapid resilience of bacterial population structures. As such, these results suggest that *Viruses* could act as moderating agents by targeting the most active populations of soil microorganisms in a “kill-the-winner” strategy^36^.

As for the other microbial groups; the wheat residue input still had a clear impact on the structure of the protist community on day 3, suggesting that copiotrophic protists (or perhaps predators of copiotrophic microorganisms) might multiply quickly following C-input into the soil. Moreover, we observed a shift of both control and amended communities from the initial state at day 125 (Fig. 1), implying that factors other than amendment might impact this group. It could be, for example, an influence of seasonality, as day 125 samples were collected in January. Indeed, RDA analyses including land use, amendment and meteorological data (i.e. we chose soil temperature at 10 cm depth, as soil humidity was strongly anticorrelated to temperature) demonstrated that the protist community was the only microbial group majorly influenced by climatic conditions (Supplementary Fig. 5 & Supplementary Table 1). These results are in agreement with a global distribution study that evidenced that meteorological data were good predictors of the composition of the protist soil community^37^.

### Microbial heterotrophic successions revealed common and specific patterns according to land-use history

**Fig. 3:**
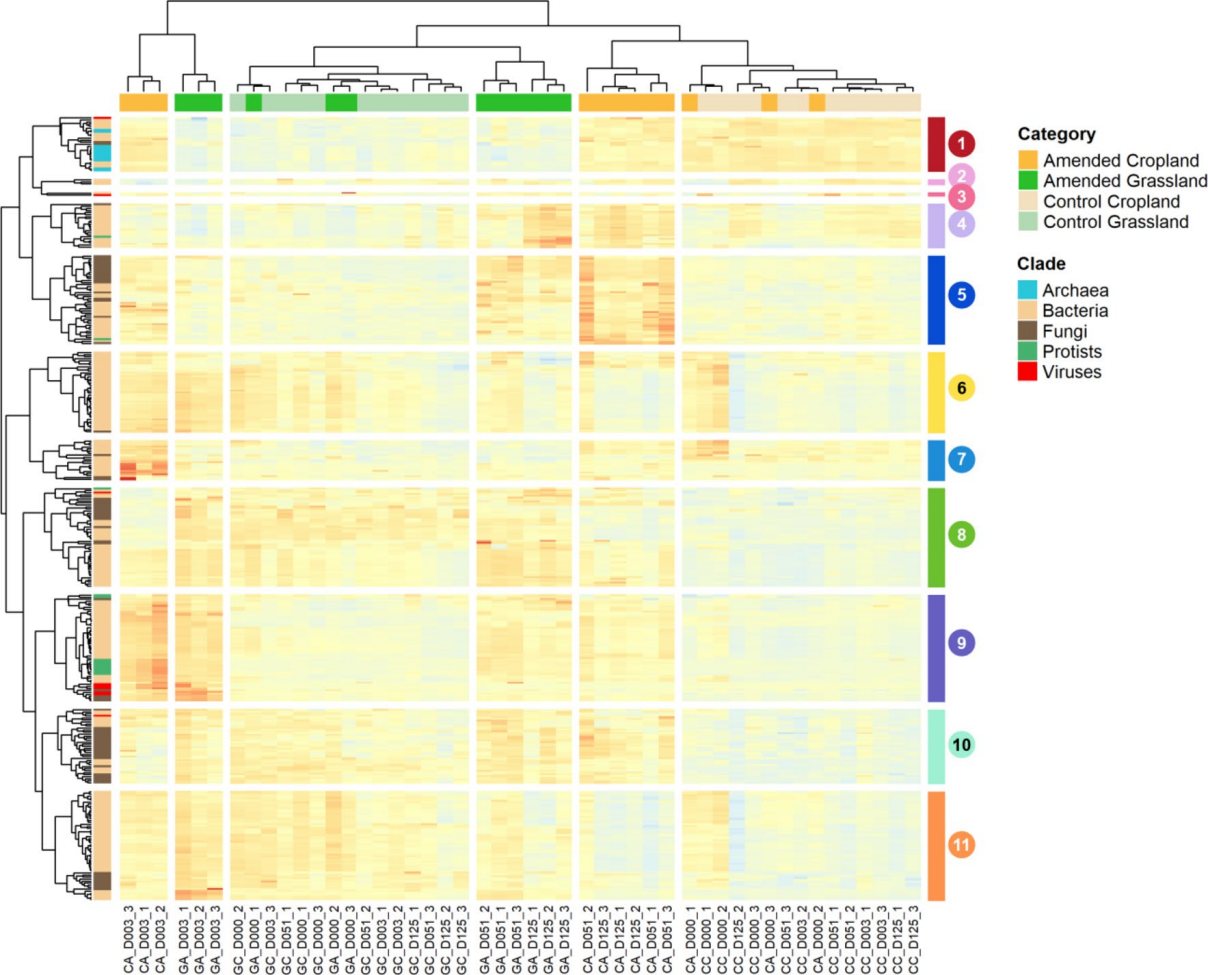
DAG and sample hierarchical biclustering. Clusters of *genera*, some specific to one land-use history, some generalist, that multiply in either the early phase or late phase after amendment are observed. Sample hierarchical clustering displayed at top, DAG hierarchical clustering on the left. DAGs clusters numbers are indicated in colours next to the *genera*, as well as their taxonomic assignation on the left.

To elucidate the abundance patterns of *genera* within the reactive part of the community, i.e., *genera* whose abundance changed significantly following wheat addition, differential analyses were performed on microbial *genera* with DESeq2, between control and amended conditions. We identified 351 Differentially Abundant *Genera* (or DAGs) from all domains of life (Supplementary Table 2). Using these DAGs, hierarchical biclustering was performed to identify the succession of populations during the whole kinetics linked with land management history (Fig. 3). In parallel, Principal Component Analyses (PCAs) and PERMANOVAs were performed on each of the *genera* clusters defined with the hierarchical biclustering to assess their role in community response^38^. This analysis evidenced different features of the response of the whole soil microbial community to wheat straw residue input.

Firstly, the clustering of the samples above all showed a separation between the amended samples 3 days after the straw input from all other samples, regardless of land management history (Fig. 3). This highlighted the strong impact of the wheat straw input on soil microbial communities in the early decomposition phase. Notably, many over-represented microbial *genera* were common to both land use histories during this early phase of degradation. These generalist *genera*, like *Massilia*, a bacterial *genera* which was also found to proliferate in other straw amendment studies^25,39^, and the fungus *Mortierella*, were mainly gathered in DAGs cluster 9. Interestingly, cluster 9 also encompassed protists such as *Pythium* and several viral *genera* of the class *Caudoviricetes* (which groups bacterial and archaeal phages) like *Amigovirus*.

Secondly, amended soils on day 3 were discriminated between croplands and grasslands, suggesting differences between the early responses of the microbial communities to the straw addition according to land-use history. Put simply, these differences stemmed from early responder *genera* reacting specifically in one land-use history with *genera* belonging to cluster 11, e.g., *Leifsonia* and *Paraburkholderia*, specific to grassland; or to cluster 7, e.g., *Serratia* and *Enterobacter*, specific to cropland).

Thirdly, all the other samples were separated according to their land-use history, with unamended soil microbiota remaining close together throughout the whole kinetic. This separation was mainly driven by cluster 1 *genera* which comprised all the archaeal DAGs which were more represented in croplands, and cluster 8 *genera* that were more abundant in grasslands. Interestingly, in these clusters, amended day 0 samples were clustered with the control samples. It was obviously due to a lack of time for the communities to respond to wheat addition, but it confirms the robustness of our field experiment and sampling approach.

Finally, days 51 and 125 amended soil samples were clustered together within their respective land-use histories. This can be linked with the response of *genera* stimulated during the late phase of wheat straw decomposition (days 51 and 125) belonging to DAGs clusters 4, 5, and 10. Again, some responding *genera* were common to both land-use histories (e.g., *Chitinophaga*,* Fusarium*, *Syncephalis*) while others were specific to grasslands (e.g., *Arenimonas*,* Cytophaga*) or croplands (e.g., *Hypoxylon*, *Lysobacter*, *Rhizopus*, *Acanthamoeba*).

Archaeal *genera*, did not react strongly to the amendment, suggesting that they were not major players in carbon decomposition. *Bacteria* represented the bulk of *genera* (240 out of 351) influenced by wheat straw input. The variety of their responses in the late or early phase, in one land-use or in both, illustrated the considerable breadth of life strategies amongst *Bacteria*. Fungal genera likewise showed a broad variety of responses after straw input, showing their ability to act as labile or recalcitrant organic matter degraders. Regarding protists, *Oomycetes* reacted in the early phase of decomposition in both land-use histories, showing their similarities with fungal lifestyles, while other protists did not react quickly to carbon addition. Lastly, most viral *genera* reacting to the amendment belonged to the *Caudoviricetes* phages. They responded positively and quickly after the straw input in both land-use histories. This suggests either that their hosts multiplied in the early stage of straw decomposition in both land-uses, or that they might infect multiple hosts.

While soil bacterial and fungal heterotrophic successions have already been described in previous studies^25,39^, our metagenomics survey provides a powerful tool for examining the concerted succession of all soil microbial actors. It allows looking further than the regulation of bacterial and fungal populations by resource availability, and investigating potential trophic regulations between all taxonomic groups^16^.

### Biotic interactions suggested

**Fig. 4:**
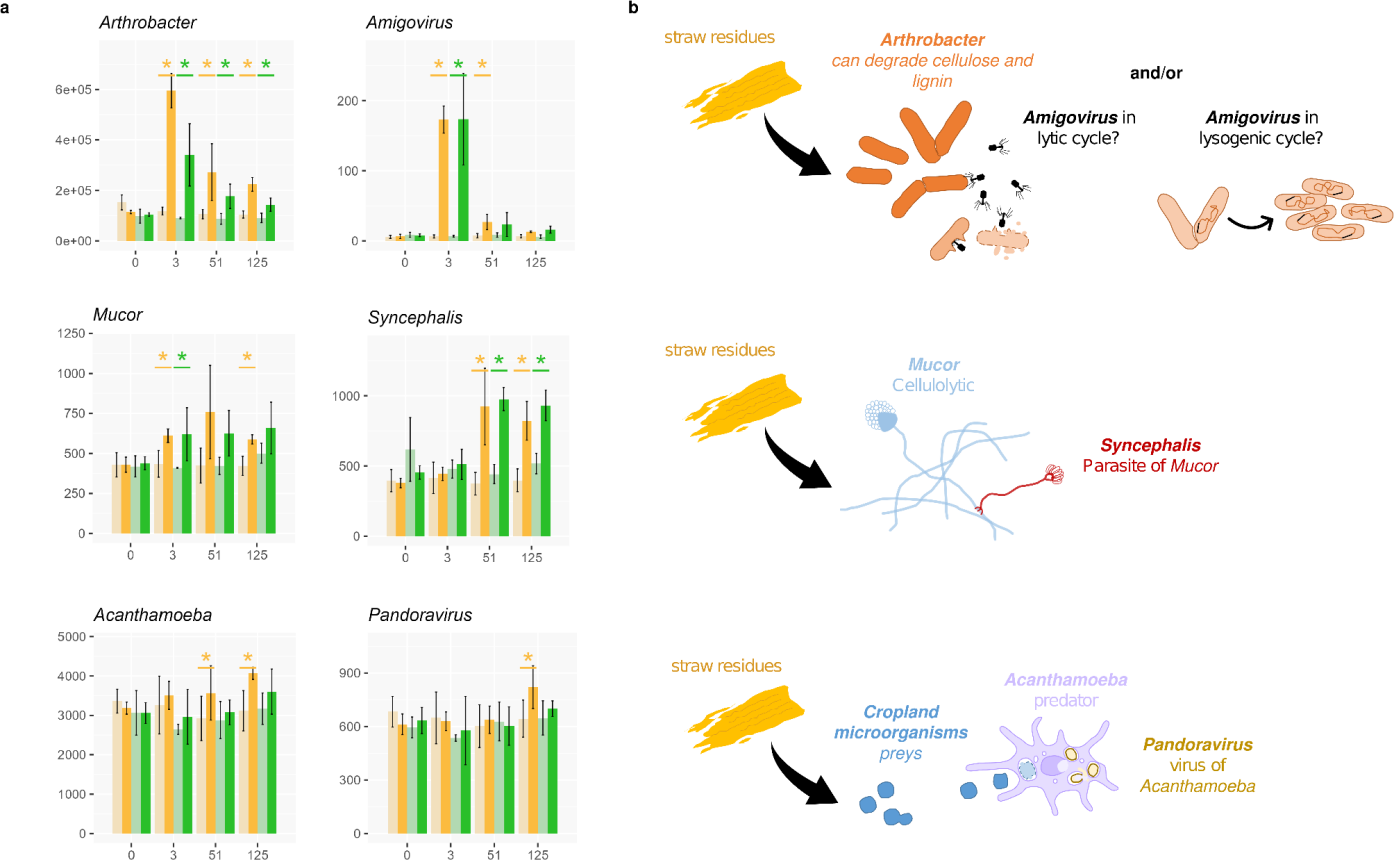
Abundance patterns of selected *genera* and theorized trophic links. Abundance profiles (**a**) of some *genera* with trophic interactions described in the literature suggest potential top-down regulation models (**b**) in our dataset. (**a**) Raw abundances of several selected *genera* along the time series. Stars denote differentially abundant counts between the control and the amended treatment at a given time point (**b**) Models of potential trophic interactions between the selected genera.

Since we looked at every microbial entity at the same time, we could hypothesise about potential interactions between taxonomic groups and how they might participate in regulating the growth of wheat straw consumers. For instance, the bacterial facultative predator *genus Lysobacter* was most abundant at day 51. *Lysobacter* species can lyse *Bacteria*, *Fungi* and *Oomycetes* cells^40^. Interestingly, all the reactive *Oomycetes genera* were found in cluster 9 (Supplementary Table 2) and were most abundant on day 3 and then decreased, perhaps in part because of predators such as *Lysobacter*. Overall, several bacterial *genera* previously characterised as facultative predator (e.g., *Cupriavidus*^41^, members of the *Cytophagales*^42^, *Ensifer*^43^, *Stenotrophomonas*^44^) were influenced positively by the straw input. However, because of their omnivorous diet, the question of whether their multiplication was fuelled mostly by wheat straw decomposition, predation or by a combination of both processes cannot be answered. In contrast, the situation was more straightforward for *Acanthamoeba*, a ubiquitous amoebozoan genus that feeds on bacterial and eukaryotic prey^45^. It was significantly more abundant in cropland amended soils at days 51 and 125, suggesting greater nutrient uptake by predation and gradual multiplication (Fig. 4). Predation by this *genus* has also been observed in a leaf litter decomposition experiment^15^. Protozoan grazers notably release the ammonium contained by their prey^46^. They have been suggested to play an important role in nutrient release from *Bacteria* to soils, where the nutrients could be exploited by other microorganisms, feeding the “microbial loop”^47^.

In DAGs cluster 9, sequences affiliated to several bacteriophages displayed significant enrichment: *Amigovirus*, *R4virus* and *Slashvirus*. Notably, *Amigovirus* is a known predator of *Arthrobacter*^48^ and sequences affiliated to this virus increased in parallel with *Arthrobacter* sequences. As *Viruses* have no means of directly exploiting wheat residues, this could suggest either a passive multiplication of viral sequences in the bacterial host, or an active lytic cycle infection (Fig. 4). Interestingly, another type of virus was found in the DAGs, namely *Pandoravirus*, a giant virus *genus* known to infect *Acanthamoeba* organisms^49^. These two organisms were most abundant specifically in croplands on day 125. We could hypothesise that populations of the predator *Acanthamoeba* could in turn be regulated by viral infection (Fig. 4). The haustorial obligate mycoparasite genus *Syncephalis* that targets *Mucorales*^50^ was found in DAGs cluster 5. This parasite might be implicated in the reduction of sequences affiliated to its potential hosts *Mucor* or *Mortierella* at days 51 and/or 125 in both land-use histories (Fig. 4). This parasitic genus was also previously shown to incorporate carbon from plant residue, alongside *Mortierella*^51^.

In conclusion, in this study we have shown that amplicon sequencing and metagenomics conducted years apart, with different technologies and with large sequencing depth differences, still provide similar assessments of in situ bacterial and fungal communities. Owing to its greater sequencing depth and its without a priori approach, metagenomics was more resolutive and allowed simultaneously assessing the whole soil microbial community (*Archaea*, *Bacteria*, *Eukaryota* and *Viruses*), hence providing precious data to decipher the interactions between groups in response to fresh plant residue inputs. Organisms from every taxonomic compartment were affected either positively or negatively by the amendment. Land-use histories impacted the soil communities and their responses to amendment, with different microorganisms responding in the two types of soils. Lastly, both inter and intra-domain trophic interactions implicating known consumer *genera* could be suggested from our dataset. Microbial predators, parasites and *Viruses* likely regulated soil communities’ response due to their generalist or specific prey/host range, thereby constituting top-down regulation and counterbalancing the bottom-up regulation of plant inputs (exudates, or plant litter)^14^.

Going further in establishing the functional potential of the soil microbiota as a whole will enable the exploration of carbon cycling pathways. In addition, by analysing the functional repertoire of the microbial community, we might gain insight into why some *genera* had different responses in the two land-use histories. For instance, it might allow distinguishing different strains with different capacities for carbon decomposition or different defence mechanisms against predators or competitors. Lastly, by analysing viral sequences more thoroughly, it could be possible to predict their potential hosts and assess whether phages are induced or integrated in their hosts’ genome. Thus, we could confirm whether, as in marine environments^52^, *Viruses* take part in a “kill-the-winner” strategy in soil environments.

## Materials and methods

### Field experiment description and soil sampling

The field experiment was conducted at Lusignan long term observatory (France), on two adjacent plots with strongly contrasted land use-histories: 20 years croplands, with crop rotation, annual tillage, herbicide crop treatment and nitrogen fertilization, or 17 years grasslands, fertilized and harvested for annual forage. The two plots’ soils had similar loamy sand textures. But some chemical properties were different between each land use: grassland soil had a mean pH of 6.02, a mean SOM of 24.05 and a mean C/N ratio of 11.38, cropland soil had a higher mean pH of 6.61, a lower mean SOM of 18.42 and a lower C/N ratio of 10.85. The detailed field experiment set up and soil characteristics were reported in^25^. Briefly, the two main plots (5 × 2 m) were prepared by manual weeding 5 month before the experiment to prevent plants’ influences on microbial communities. After weeding, each plot was divided into six 0.7 × 0.7 m sub-plots and the site was left for 5 months to stabilize, with manual weeding every week to remove regrowth seedlings. In September 2011, all sub-plots had their first 10 cm excavated, mixed and returned in place. In half of them, wheat straw residues were amended into the mixed soil (250 g per sub-plots, corresponding to 5 t dry matter ha^− 1^), while the remaining plots, serving as control, were put back in place without amendment. Four sampling dates were analysed: at the date of wheat straw amendment (T0), then 3, 51 and 125 days after amendment (respectively T3, T51, T125). It corresponded to a total of 48 soil samples (3 replicates×2 treatments×2 plots×4 dates). These sampling dates have been selected from the global sampling survey, as the community changes peaked after 3 days, and resilience of the community structure occurred more rapidly for bacteria (51 days) than for fungi (125 days)^25^.

### DNA extraction and sequencing

For metagenomics, the DNA extraction was performed using a single procedure standardized by the GenoSol platform, based on the standardized ‘ISO-11063: Soil quality Method to directly extract DNA from soil protocol^27,53^. Generally, soil DNA extraction is impaired by the complex matrix characteristics which lower target organisms’ accessibility, and/or by differences in target organisms’ cell wall composition and structure which reduce cell lysis efficiency, as observed for bacterial strains. This is particularly the case for microeukaryotes with fortified cell walls or very resilient cell membranes^54^, and for viral particles with specific capsid structure. However, the DNA extraction procedure used in this study combines a mechanical, thermal and chemical lysis step, giving a higher access to the DNA of underestimated microbial groups^27^. Library preparation and sequencing were performed by the GeT-PlaGe platform (Auzeville, France). As the DNA concentration were low, libraries were prepared using the TruSeq Nano DNA, which included a whole metagenome amplification step. Libraries were then sequenced with Illumina NovaSeq 2 × 150 bp technology.

For amplicon sequencing, the dataset originally from Tardy and collaborators^25^ was reused. Briefly, a 16 S rRNA gene fragment (targeting Bacteria and Archaea) and a 18 S rRNA gene fragment (targeting Fungi) were amplified by PCR using primers F479 & R888 and primers FR1 & FF390 respectively, following the PCR conditions described previously^25^. PCR products were then purified and pyrosequenced with a GS FLX Titanium (Roche 454 Sequencing System).

### Bioinformatic analyses

After sequencing, an average of 60.6 million read pairs were obtained for each library, representing a total of 2.9 billion read pairs, all 48 samples combined (2.14 TB for the entire dataset, with a mean GC content of around 63%). The quality control of the metagenomics shotgun paired-end reads was performed with Fastqc v0.11.9 and MultiQC v1.7. As the quality was good (PhredScore over 35 throughout the reads, no N content, no adapters, etc.), paired-end reads were used directly for taxonomic assignation, with no cleaning step. Taxonomic assignation was performed on the paired-end reads with Kaiju v.1.7.0, default parameters^55^, against the nr_euk database (v 2018). Of all reads: 0.98% were classified as Archaea, 68.01% as Bacteria, 0.72% as Eukaryota, 0.03% as Viruses, 30.26% were unclassified. Counts for a total of 57 361 different taxa were retrieved after the assignation. These metagenomic taxonomic counts were used for downstream analysis.

The amplicon dataset was initially treated with a pipeline called GnS-PIPE and developed by the GenoSol platform (INRA, Dijon, France)^25^. More precisely, the sequences were cleaned, clustered and taxonomically assigned with dedicated parameters^25^. The amplicon sequencing data was then manually corrected to account for few changes in taxonomic classification since the initial study (e.g., some taxa originally classified as Fungi *incertae sedis* were moved to other *phyla*) (modification files available at^56^).

### Statistical analyses

Statistical analyses were conducted using R v4.3.1 in the RStudio software v2023.9.1.494. Plots were generated with ggplot2 v3.4.3 unless stated otherwise. The RMarkdown script used is available at^38^.Taxonomically assigned counts were used as a measure of taxa abundances. They were converted as phyloseq (phyloseq v1.44.0) objects for easier analysis. Rare taxa detected in less than 10% of samples (representing 21.33% of the taxonomic dataset), which might be attributed to spurious assignation and might constitute noise in the data, were excluded from further analysis. 45 127 taxa were kept for downstream analyses.

Three normalisation methods were tested to remove the biases induced by differences in library size and sampling fractions^57^. First, the traditional rarefaction, where filtered taxonomic counts were randomly subsampled at the depth of 95% of the smallest library for each large taxonomic group. Common limitations of this approach evoked in the literature are a loss of valid information and an introduction of an additional sampling bias^57^. Second, the Centred Log-Ratio (CLR) transformation was performed on the filtered taxonomic counts (microbiomeMarker v1.6.0). Developed to deal with compositional data such as microbiome data^58^ the CLR transformation centres the data and eliminate library size biases. Negative values in the CLR taxonomic counts matrix were set as zero, considering that these very low abundance counts were negligeable. Lastly, the ANCOM-BC normalisation procedure was tested on the filtered counts data (ANCOMBC v2.2.2). Also based on log-ratio this method has a bias correction procedure. The results of these three different normalisation techniques can be observed in a RMarkdown report^38^. On a computation time stand-point, with our 45 127 taxa dataset, both rarefaction and ANCOM-BC ran slowly (1118.75 s and 5389.42 s respectively, running time measured with package tictoc v1.2.1). In contrast the CLR transform ran far quicker (0.38 s). The normalised datasets obtained after each of the three procedures were then used to compute Principal Coordinates Analyses (PCoA) using Euclidean distance (equivalent to an Aitchison distance for our log-ratio transformed datasets^59^) with phyloseq v1.44.0. Both the CLR and ANCOM-BC methods allowed for a clear separation of samples according to their land use histories for every taxonomic group, while rarefaction was less resolutive, notably for Fungi and Viruses. Total explained variability tended to be higher for the CLR data than the ANCOM-BC data. Based on these results, the CLR normalisation procedure was chosen, it performed well in term of computation time for our taxa-rich dataset, allowed for cropland and grassland communities separation, was more straightforward and better established in the literature than ANCOM-BC. Permutational Multivariate Analysis of Variance (PERMANOVA, 999 permutations) was used to estimate the significance of observed differences between groups (vegan v2.6-4).

To estimate the potential influence of technical or environmental variables on soil microbial communities, climatic data collected on site and DNA libraries properties were screened to select relevant and non-colinear variables with corrplot v0.92 and PerformanceAnalytics v2.0.4. Kept quantitative variables were scaled and the best variance partitioning model of the whole CLR normalised community data was computed, using phyloseq and vegan. This model (microbiome_data ~ wheat_straw_amendment + land_use + soil_temperature) was then used to compute RDA for each microbial group taxonomic composition.

Cooccurrence networks for each land use history X treatment combination were built using the statnet suite v2019.6, including ergm v4.6.0. For each category, all the time points were grouped together, amounting to 12 samples. Repeated network generation was performed, with each one of the 12 samples left out in turn. The metrics of the 12 repetitions per category were taken into account for the analysis.

Differential analyses were performed with DESeq2 v1.40.2 on the raw, unfiltered taxonomic counts data, agglomerated at the *genus* level (with phyloseq v1.44.0), to detect potential DAGs (padj threshold: < 0.01, log2FoldChange threshold: >0.25). For each land use and time point, the amended samples were tested against the corresponding control samples, to remove potential seasonal effects (temperature and precipitations variations notably). The resulting DAGs were then filtered according to their prevalence in their respective domain (only those representing at least 1% of their domain were kept for further exploration). A CLR transformation was performed on the data agglomerated at the genus level, with again negative values set to zero and a rare *genus* (detected in less than 10% of samples after the transformation and negative removal) filter was applied. A biclustering of the samples and the filtered DAGs was plotted using the CLR transformed data agglomerated at the genus level, and Pearson distance (corresponding to 1 – Pearson correlation) with ComplexHeatmap v2.16.0. The DAGs that did not pass the CLR filters were not included (28 out of 379, leaving 351 *genera*).The resulting clusters of DAGs were then separately analysed by PCA and pairwise PERMANOVA (pairwiseAdonis v0.4.1), to detect on which grouping variable the samples were separated. Lastly, the raw abundances of each DA*G*s were plotted to better grasp their dynamics in the experiment.

Amplicon sequencing data (1.18 MB for the entire dataset) was reused from^25^, with as normalisation procedure, a rarefaction by random sampling to a level of 4000 reads for 16 S rRNA gene data (0.05% of reads assigned to Archaea, 92.17% to Bacteria and 7.77% unclassified) and 8000 reads for 18 S rRNA gene (39.36% of reads assigned to Fungi, 60.64% unclassified). No other normalisation procedure was performed. PCoA of the communities separated by domain were performed, as well as differential analyses with DESeq2 as described for the metagenomic data. Raw abundance profiles for *genera* of interest were plotted. The complete workflow can be viewed here (Supplementary Fig. 6).

## Electronic supplementary material

Below is the link to the electronic supplementary material.

Supplementary Material 1

Supplementary Material 2

Supplementary Material 3

Supplementary Material 4

Supplementary Material 5

Supplementary Material 6

Supplementary Material 7

Supplementary Material 8

Supplementary Material 9

## Data Availability

The raw read datasets were deposited at DDBJ/ENA/GenBank under the BioProject accession number PRJNA1159878^60^. The taxonomic counts as well as the metadata and the statistical analyses performed for the current study are available in a Recherche Data Gouv repository (10.57745/OMJUXC, 10.57745/HXN1XT, 10.57745/RXJXHW).
