# Supplementary material for: Metagenomics reveals contrasted responses of microbial communities to wheat straw amendment in cropland and grassland soils
Source: Sci Rep. 2025 Apr 27;15:14723. doi: 10.1038/s41598-025-98903-2 (PMC12034817; doi:10.1038/s41598-025-98903-2)
Supplement: Supplementary file 9 — Supplementary Material 9 [file 41598_2025_98903_MOESM9_ESM.docx]

**Supplementary Figure 1: PCoA of communities separated by taxonomic group, land-use history and treatment.** Most amended soil communities significantly differ trough time, in contrast to control communities that retain similar structures. This is observed for both metabarcoding and metagenomics. **a.** PCoA of the metabarcoding data. **b.** PCoA of the metagenomic data. Percentages of explained variance are indicated in square brackets. The *p*‑values of PERMANOVAs between time points are indicated in parentheses.

**Supplementary Figure 2: Major *phyla* relative abundances for each sample measured with metagenomics and metabarcoding**. Bacterial and fungal *phyla* relative abundances were similar with both approaches. **a.** Bacterial phyla relative abundances. **b.** Major fungal *phyla* relative abundances. *Phyla* representing less than 1% of the total bacterial or fungal counts in every control day 0 samples were placed under “other”. Candidate *phyla* were also placed under “other”.

**Supplementary Figure 3**: **All bacterial *phyla* relative abundances for day 0 samples detected with metagenomics and metabarcoding**. Many low abundance bacterial *phyla* were exclusively detected by metagenomics.

**Supplementary Figure 4**: **Abundance comparison of *genera* previously highlighted**. Both metabarcoding (**a** and **b**) and metagenomics (**c** and **d**) produced similar abundance patterns at the *genera*-level.**a.** Raw metabarcoding abundance of genera presented in ^25^. **b.** Raw metagenomic abundance of the same genera if available. Stars denote differentially abundant counts in the amended condition compared to the corresponding control condition.

**Supplementary Figure 5: RDA of communities separated by taxonomic group.** Protistan communities were strongly influenced by temperature in contrast to the other microbial communities. Percentages of explained variance are indicated in square brackets. The p-values of Permutation Tests for each axis are indicated in parentheses.

**Supplementary Figure 6:** **Chosen bioinformatic workflow.** The taxonomic counts were obtained directly from unassembled raw reads.

**Supplementary Table 1: RDA summary results for each microbial group.** In contrast to other microbial groups that were firstly affected by land-use, protists communities structure variance was explained firstly by soil temperature. The proportion of explained variance and the adjusted *p*-value is given for each chosen explanatory variable (land use, amendment, temperature) and each separated microbial group. Significant p-values are indicated in bold.

**Supplementary Table 2: DAGs identified between control and amended conditions.** DAGs from all domains of life were found in response to amendment. Taxonomy, assigned DAGs cluster and manual annotations are indicated.
